# Spelling Impairments in Italian Dyslexic Children with and without a History of Early Language Delay. Are There Any Differences?

**DOI:** 10.3389/fpsyg.2016.00527

**Published:** 2016-04-19

**Authors:** Paola Angelelli, Chiara V. Marinelli, Marika Iaia, Anna Putzolu, Filippo Gasperini, Daniela Brizzolara, Anna M. Chilosi

**Affiliations:** ^1^Department of History Society and Human Studies - Lab of Applied Psychology and Intervention, University of SalentoLecce, Italy; ^2^IRCCS Foundation Santa LuciaRome, Italy; ^3^IRCCS Foundation Stella MarisPisa, Italy; ^4^Department of Developmental Neuroscience, University of PisaPisa, Italy

**Keywords:** developmental dyslexia, developmental dysgraphia, shallow orthographies, phonological processing, early language delay, spelling errors

## Abstract

Language delay is considered a frequent antecedent of literacy problems and both may be linked to phonological impairment. However, while several studies have examined the relationship between language delay and reading impairment, relatively few have focused on spelling. In this study, spelling performance of 28 children with developmental dyslexia (DD), 14 children with a history of language delay (LD), and 14 children without (NoLD) and 28 control participants were examined. Spelling was investigated by a writing to dictation task that included orthographically regular stimuli (word and non-words), as well as words with unpredictable transcription. Results indicated that all dyslexic participants underperformed compared to controls on both regular and unpredictable transcription stimuli, but LD performance was generally the worst. Moreover, spelling impairment assumed different characteristics in LD and NoLD children. LD children were more sensitive to acoustic-to-phonological variables, showing relevant failure especially on stimuli containing geminate consonants but also on polysyllabic stimuli and those containing non-continuant consonants. Error analysis confirmed these results, with LD children producing a higher rate of phonological errors respect to NoLD children and controls. Results were coherent with the hypothesis that among dyslexic children, those with previous language delay have more severe spelling deficit, suffering from defective orthographic lexical acquisition together with long-lasting phonological difficulties.

## Introduction

A high incidence of language impairment in preschool years has been found in children with developmental dyslexia (DD) both retrospectively (Kinsbourne and Warrington, 1963; McArthur et al., 2000; Snowling et al., 2003; Brizzolara et al., 2006; Chilosi et al., 2009) and prospectively (Scarborough, 1990; Leppänen et al., 2010). Moreover, in DD children, weaknesses in different expressive and receptive oral language measures at school age concurrent with written language difficulties have also been documented (e.g., Stark and Tallal, 1988; McArthur et al., 2000; Chilosi et al., 2009). On the other hand, a large body of evidence sustains that children with Specific Language Impairment (SLI) are at risk for development of reading and spelling difficulties at school age (e.g., Brizzolara et al., 1999; Goulandris et al., 2000; Bishop and Snowling, 2004).

However, links between oral and written language are complex and it is still unclear which specific linguistic deficits, responsible for reading and spelling difficulties, are shared by SLI and DD children.

For some authors, SLI and DD subjects have difficulties in tasks requiring fine-grained phonological skills, such as metaphonological and non-word repetition tasks (Brizzolara et al., 1999; Snowling, 2000; Bishop and Snowling, 2004; Ramus et al., 2013). Phonological abilities are considered crucial for acquisition of correspondences between letters and sounds, the foundation of reading in alphabetic systems (Ramus et al., 2003). Difficulties in analysing linguistic units on a subsyllabic level may hamper acquisition of grapheme–phoneme and phoneme–grapheme correspondences (Bird et al., 1995) and these difficulties may be common in SLI children and in those with DD. In this framework, most studies have focused their attention to the relationship between oral language abilities (mostly phonological deficits) and reading difficulties (Catts, 1993; Snowling, 2001; Ramus et al., 2003) while a few have considered also spelling (e.g., Bishop and Clarkson, 2003; McCarthy et al., 2012). Spelling is a very demanding and sensitive task, which may reveal minor problems that might otherwise go undetected. Spelling errors may result from residual problems in adults with compensated dyslexia (i.e., Bruck, 1993) as well as in adult relatives of dyslexic children (i.e., Wolff et al., 1996). Moreover, spelling may provide a deep examination into orthographic and phonological skills also in a more consistent language such as Italian, contributing to the debate on the role of orthographic and phonological processing in literacy-acquisition deficit (McCarthy et al., 2012). Spelling, in fact, depends on efficiencies of both phonological and lexical processes, as well as their interaction. Following dual-route models (e.g., Patterson, 1986; Hillis and Caramazza, 1991; Kreiner, 1992; Barry, 1994; Perry et al., 2002), learning to spell consists of progressively mastering two strategies: lexical procedure, which relies on accessing word-specific memory, and a sublexical procedure which relies on exploiting regularities in sound-to-spelling (e.g., phoneme–grapheme) correspondences. Deficits or difficulties on lexical or semantic levels may be detected by spelling difficulties of irregular or unpredictably spelled words (e.g., phonological strings that may have multiple orthographic solutions). Note that in Italian, as in most regular orthographies, there is a certain degree of ambiguity in oral-to-written direction and there are several instances of unpredictable spelling: for instance, the phonemic group [kw] may be transcribed by the orthographic sequences QU or CU (Angelelli et al., 2004, 2010a).

Regarding sublexical process, phonemic strings must first be identified and segmented by an acoustic-to-phonological conversion unit. Then, graphemes corresponding to plausible spelling are activated by means of a phoneme-to-grapheme conversion process that uses sound-to-spelling correspondences (Patterson, 1986). Acoustic-to-phonological analysis is subject to certain variables, which determine complexity such as phonetic-acoustic quality of phones. It is easier to isolate and identify continuant phones, i.e., vowels and fricatives ([f], [v], [s], [ʃ] liquid [l], [r]) and nasal consonants ([n], [m], [ɲ], [ŋ]) that are susceptible to prolongation. Similarly, it is easier to analyze words containing consonant–vowel sequences (CV) than words containing consonant clusters (e.g., senso, [‘sεnso], sense or valle, [‘val:e], valley). Defective analysis of phonemic strings cause incorrect spelling even if phoneme-to-grapheme conversion is unimpaired. In Italian patients with acquired dysgraphia, it is quite common to find intact phoneme-to-grapheme conversion skills (writing of single letters) with impaired isolation and identification of each single phoneme within a phonemic sequence to be converted (Luzzatti et al., 2000).

Reliance on sublexical procedure is prevalent in children learning in transparent orthographies. In fact, cross-linguistic studies indicate that acquisition of different procedures is not the same across languages and depends on degree of orthographic consistency of different languages (Caravolas, 2004; Sprenger-Charolles et al., 2006). The more regular the writing system, the more children rely on sublexical processing. A recent study on development of spelling skills in first- to eighth-grade Italian normal readers (Notarnicola et al., 2012) confirmed prevalent reliance on phonological procedure, even though signs of lexical spelling were detected since early grades. Moreover, in languages with shallower orthography, phonological factors may play a more prominent role in spelling than in reading. In a series of longitudinal studies on children learning Turkish, a shallow language, Babayiğit and Stainthorp (2007, 2011) first demonstrated that preschool phonological awareness failed to make any reliable contribution to future reading skills, but was the strongest longitudinal correlate of spelling skills measured at the end of Grades 1 and 2. In a second study, Babayiğit and Stainthorp (2011) confirmed that phonological awareness was the strongest predictor of spelling, while Rapid Automatized Naming (RAN) was a powerful predictor of reading fluency for school-aged children. Overall, the findings suggested that phonological awareness may be differentially related to reading and spelling, and that spelling is a more sensitive index of phonological processing skills.

Similar results are provided from studies on children with developmental dysgraphia and dyslexia. In a longitudinal study on German-speaking children, Wimmer and Mayringer (2002) found retrospectively that only third-grade children with a single spelling deficit had poor phonological awareness and phonological short-term memory in school-entrance assessment. In contrast, children with isolated reading fluency deficits had difficulties in RAN tasks, but did not presented phonological deficits. Therefore, it was concluded that only a specific spelling deficit is preceded by oral phonological difficulties.

Regarding Italian, a series of studies on DD children have specifically investigated characteristics of spelling impairments (Angelelli, 2004; Angelelli et al., 2004, 2010a). In these studies, a psycholinguistic approach was adopted in order to specifically test efficiency of lexical and sublexical spelling processes. To achieve this, spelling tasks included regular stimuli (words and non-words), as well as words with unpredictable transcription. All regular stimuli were selected with different sources of phonetic-phonological difficulties in order to exploit efficiencies of acoustic-to-phonological analysis of spelling process. By contrast, words with unpredictable transcription more selectively mark lexical-orthographic procedure. Moreover, error analysis was adopted to identify nature of spelling errors (lexical-phonologically plausible errors vs. phonological ones, i.e., inaccurate spellings via sublexical procedure), since analysis of errors can shed light on underlying dysfunctional mechanisms.

Particular attention was given by the authors to phonological errors consisting in minimal distance substitutions (i.e., substitutions of consonants or vowels with other consonants or vowels that differs only in one single distinctive feature): such misspellings may be ascribed to mild impairment of acoustic-to-phonological conversion (Luzzatti et al., 2000; Georgiou et al., 2010; Leppänen et al., 2010). Both accuracy and error analysis indicated that younger DD subjects (3rd graders) suffered from an inefficiency of both sublexical and lexical processes with inaccurate spelling of both regular and unpredictable spelling stimuli and a high rate of all types of errors. Older dyslexic children (5th and 6th graders), instead, presented a marked lexical spelling deficit with difficulties mainly confined to stimuli with unpredictable spelling and prevalence of phonologically plausible errors. The authors hypothesized that with school progression, given the relatively transparent orthography of Italian language and a phonic teaching method, DD children will persist in spelling via phonological encoding, so they can overcome initial difficulties through sublexical spelling procedure more easily than through the lexical one. However, cases differing from this trend were also observed, showing a persistency in phonological difficulties in spelling (Angelelli, 2004; Angelelli et al., 2010a) that were ascribed to other potentially relevant factors such as concomitant deficits of phoneme discrimination, processing, and representation.

In this view, the study of spelling performance of DD children who have experienced a previous language delay may contribute to understanding if specific spelling error patterns may be ascribed to residual subtle phonological and language difficulties.

Two studies have analyzed reading and spelling performance of Italian DD children with (LD) and without (NoLD) a history of language delay in preschool years aimed at understanding if Italian children with DD showed selective phonological processing deficits (Chilosi et al., 2003, 2009) or more widespread linguistic impairments (Chilosi et al., 2009). In these studies, no differences between LD and NoLD children were found in severity of reading speed deficit. However, LD dyslexics tended to be more inaccurate in text comprehension than NoLD children.

Regarding spelling, results were controversial. In the first study, LD dyslexics were found to be more impaired than NoLD dyslexics in spelling under dictation of a short passage and lists of words and non-words. This result however was not replicated in the second study where no differences in spelling accuracy were detected. In this second study, LD children were more impaired in phonological-working memory, phonological fluency, as well as in semantic fluency, grammatical comprehension, and verbal IQ. Overall data supported presence of moderate but widespread linguistic deficits (not limited to phonological processing) in a subset of DD children with previous language delay that is not generalized to all children with reading difficulties. It is worth noting that spelling tasks used in the above studies were not sufficiently sensitive to detect different deficits along lexical and sublexical procedures and a qualitative analysis of errors was not performed. In fact, stimuli varied only for lexicality and did not explore the effects of other relevant psycholinguistic and acoustic-to-phonological variables.

In this study, we investigated spelling skills of DD children with LD and NoLD, using stimuli that varied not only for lexicality and regularity of transcription but also for different sources of acoustic-to-phonological complexity such as continuance of sounds, length, and presence of doubled consonants. If only dyslexic children with LD suffered from phonological processing deficits, we would expect differential effects of these specific variables in LD respect to NoLD dyslexic children. Moreover, a qualitative analysis of errors was performed to obtain further information on possible loci of spelling difficulties. Coherent with the hypothesis of long-lasting phonological difficulties in LD children, a prevalence of errors indicating failure in acoustic-to-phonological analysis would be expected. To our knowledge both spelling analyses have not been previously performed in such a population.

In conclusion, we expected both groups of DD children to suffer from a lexical spelling deficit (Angelelli et al., 2004, 2010a), but only DD with LD would show concurrent relevant sublexical spelling deficits.

## Methods

This study was made possible thanks to an agreement between the Department of Psychology of the University of Bari and the “Stella Maris” Foundation—Department of Developmental Neuroscience—University of Pisa. All participants were natives to areas in and around Bari and Pisa. They were referred to our psychology and neuropsychology units for suspected reading impairments. In accordance with the discrepancy definition (American Psychiatric Association, 2013), criteria for inclusion were a marked reading delay on a standard reading task (see Section Verbal Abilities Assessment) associated with normal intelligence measured by Raven's Progressive Matrices (performance within 2 *SD*s according to the norms); (Pruneti et al., 1996) and normal socio-educational conditions. The final sample included 28 developmental dyslexic children (DD) (17 males and 11 females), ten of which were in third grade, fourteen in fourth grade and four in fifth grade. Age ranged from 8.1 to 11.1 years (*Mean* = 9.4, *SD* = 0.79).

Each child's clinical history was investigated by means of an assessment interview with parents. This was carried out by a child psychologist with expertise in clinical neuropsychology. Parents were also asked to fill out a questionnaire (Chilosi et al., 2003, 2009; Brizzolara et al., 2006) on motor, cognitive, and language developmental milestones. In order to encourage parents to recall basic language milestones, examples of typical child utterances were provided. All questionnaires were checked by an independent rater, a child neuropsychiatrist experienced in speech and language pathology (A.C), who did not participate in any further testing. Children were considered to have a history of language delay (LD) if analysis of their questionnaire showed at least two of the following signs: (1) no vocabulary burst before 24 months; (2) late combinatory use of words (that is, after 30 months); (3) persistent grammatically incomplete sentences after 4 years of age, and (4) persistent phonological mispronunciations after 4 years of age.

According to these criteria, 14 children (6M, 8F) were identified as having a history of language delay (LD) with a mean age of 9.33 years (*SD* = 0.65 months) and an average Raven test of −0.24 (*SD* = 0.66). No language delay (NoLD) was documented retrospectively in 14 children (11M, 3F) with a mean age of 9.52 years (*SD* = 0.92 months) and an average Raven test of −0.86 (*SD* = 0.79). There were no significant differences in age [*F*_(1, 27)_ = 0.38, ns], cognitive level [*F*_(1, 27)_ = 2.84, ns], or gender distribution (Yates corrected *X*^2^ = 2.4, ns) between LD and NoLD groups.

The two groups of DD children were compared to a control group of 28 normal readers (17 M, 11 F, mean age = 9.48, *SD* = 0.78; mean Raven performance = –0.49, *SD* = 0.75) matched for 1 to 1 for gender as well as for age [*F*_(2, 53)_ = 0.22, ns] and cognitive level [*F*_(2, 53)_ = 0.03, ns].

Parents were informed of screening activities and authorized their child's participation by signing the appropriate consent form. The study was conducted according to the principles of the Helsinki Declaration and was approved by the local committee of the Departments and school authorities.

### Reading assessment

Participant reading level was assessed by a standard reading test widely used for Italian children (*MT Reading Test*, Cornoldi et al., 1998). This test was used as inclusion criterion for each group. Two meaningful text passages were presented: the first evaluating decoding abilities, the second comprehension skills. Participants were asked to read the first passage aloud (within a 4-min time limit). Two parameters, speed (time in seconds per syllable) and accuracy (number of errors, adjusted for amount of text read) were considered. Raw scores were converted to *z*-values according to standard reference data (Cornoldi et al., 1998). DD children scored at least 2*SD*s below mean score of normative sample for either reading speed or accuracy. A selective failure either in speed or accuracy was sufficient to satisfy inclusion criteria, because children with reading problems may modify their ability to read faster strategically (with loss of accuracy) or more accurately (at the expense of speed; Hendriks and Kolk, 1997; Di Filippo et al., 2006). Control children performed within reference norms for both reading speed and accuracy.

The second passage, evaluating reading comprehension, was given without any time limit. Participants had to read it and respond to 10 multiple-choice questions (measure of comprehension). Raw scores were converted to *z*-values according to reference data (Cornoldi et al., 1998). Nature of reading disturbance of DD participants was also examined by additional tasks. In particular, single word and non-word reading was assessed by means of *Developmental Dyslexia and Dysorthography Battery* (Sartori et al., 1995). The word list consists of four groups of 28 high and low-frequency words, varying in length from 4 to 8 letters. The non-word list consists of 48 non-words varying in length from 5 to 9 letters. Number of errors and speed of reading (syllables/s) were scored.

Mean *z*-scores (and standard deviation) for speed, accuracy, and comprehension of reading test (Cornoldi et al., 1998), as well as single word and non-word reading speed and accuracy (Sartori et al., 1995) are given in Table 1 for both groups of DD children. For each reading parameter, one-way ANOVAs comparing group performance were carried out.

**Table 1:** **Mean *z*-scores (and *SD*) on reading skills of normal readers and both LD and NoLD dyslexic children**.

	**Normal readers**		**NoLD dyslexic children**		**LD dyslexic children**		**LD vs. NoLD dyslexic children**	
	***Mean***	***SD***	***Mean***	***SD***	***Mean***	***SD***	***F*_(1, 27)_**	***p***
**TEXT READING**								
Speed	0.05	0.45	–1.11	1.1	–2.12	2.32	2.16	0.15
Errors	–0.28	0.59	–3.31	1.81	–3.75	1.99	0.37	0.54
Comprehension	–0.02	0.54	–0.70	0.75	–0.57	0.67	0.20	0.66
**SINGLE STIMULUS READING**								
Word: speed	–0.42	0.66	–1.08	1.51	–1.99	2.23	1.06	0.32
Word: errors	0.79	1.31	–4.53	3.35	–5.38	3.02	0.30	0.60
Non-word: speed	–0.35	0.55	–0.65	1.21	–0.93	1.78	0.15	0.70
Non-word: errors	0.82	0.99	–3.07	2.00	–2.48	1.17	0.50	0.49

LD and NoLD groups had similar performance in all reading parameters, in both text passage and single word and non-word reading. Note that, in general, in the present sample of DD children, reading deficits seemed more severe for accuracy than speed, for both passage and single-word and non-word reading. Word and non-word reading tests highlighted a more severe deficit for words with respect to non-words in both groups of DD children. Moreover, neither group showed a severe deficit in text comprehension.

Overall, as expected on the basis of inclusion criteria, both DD children with LD and NoLD showed marked deficits in reading a meaningful passage as well as in single words and non-words. However, no significant differences emerged between groups regarding severity/characteristics of reading deficit.

### Verbal abilities assessment

Although, none of DD participants showed overt signs of language impairment on a discourse level, a brief battery of verbal abilities was also administered in order to check current verbal abilities. Verbal functioning assessment was also extended to control participants in order to take into account potentially relevant variables. Battery included: (i) a test of phonological working memory consisting of a repetition of increasing longer lists (from 2 to 6 words) of disyllabic phonologically similar and non-similar words, upon computerized acoustic presentation (Brizzolara and Casalini, 2002). For each list, memory span was calculated as the number of longest sequences correctly repeated in at least three out of five presentations; (ii) a receptive vocabulary test (Peabody Picture Vocabulary Test; Dunn and Dunn, 1997; Italian version by Stella et al., 2000), in which subjects were asked to select, among four illustrations, the picture that represents the meaning of a word orally presented by examiner; (iii) an expressive vocabulary test (Picture Naming Test; Brizzolara, 1989), in which subjects were asked to name 104 pictures corresponding to high (52) and low (52) frequency words. Due to technical problems, the working memory test was administered to 10 out of 14 NoLD children and the expressive vocabulary test to 13 out of 14 NoLD children.

For all verbal tests, raw scores were converted to *z*-scores according to reference data. Results are reported in Table 2. For each linguistic measure, one-way ANOVAs comparing group performance (LD, NoLD, control participants) were performed (see right side of Table 2 for significance of main effects). Bonferroni *post-hoc* comparisons were used to explore significant main effects.

**Table 2:** **Mean z scores (and *SD*) on linguistic skills of three groups of participants**.

	**Normal readers**		**NoLD dyslexic children**		**LD dyslexic children**		***F***	***p***	**Group differences**
	***Mean***	***SD***	***Mean***	***SD***	***Mean***	***SD***			
**WORKING MEMORY**									
Phonologically non-similar disyllables	–0.42	0.95	–0.76	1.18	–1.50	1.27	4.57	0.02	DL < NoDL = Control
Phonologically similar disyllables	–0.62	1.10	–1.71	1.00	–2.19	1.57	8.34	0.00	DL < NoDL = Control
**RECEPTIVE LANGUAGE**									
Peabody picture vocabulary test	0.18	0.86	–0.76	0.90	–0.80	1.05	7.65	0.00	DL = NoDL < Control
**EXPRESSIVE LANGUAGE**									
High-frequency words	–1.49	1.17	–1.28	1.52	–1.69	1.55	0.31	0.74	DL = NoDL = Control
Low-frequency words	–1.30	0.76	–1.74	0.99	–2.27	1.01	5.72	0.01	DL < NoDL = Control

*F-values (and relative p) refers to one-way ANOVA comparing the three groups in each reading parameter. The column group differences indicate differences between groups as reported in Bonferroni post-hoc test. Note: symbol = refers to non-significant differences between groups, symbol < to a worse performance of the preceding group with respect to the following one*.

As seen from Table 2, for the phonological working memory test, both DD groups had a lower performance for phonologically similar words than non-similar ones, although LD children seemed more affected.

A main effect of *group* was found. *Post-hoc* comparisons revealed that LD participants were more impaired than normal readers for both phonologically similar words (*p* < 0.001) and non-similar ones (*p* < 0.01). Instead, NoLD children revealed no differences from control group. Concerning task with phonologically similar words, LD group also performed worse with respect to NoLD children (*p* < 0.05).

For receptive vocabulary test, *z*-scores showed marginal deviation from test norms in both groups of DD children. ANOVA showed a main effect of *group*, with DD children generally underperforming with respect to control participants but no differences emerged between LD and NoLD. *Post-hoc* comparisons revealed that both groups of DD children performed poorly with respect to normal readers (at least *p* < 0.01).

In reference to expressive vocabulary test, for high-frequency words no significant differences emerged between groups, while a group effect was evident for low-frequency words. *Post-hoc* comparisons showed that only LD children were more impaired than normal readers (*p* < 0.01). NoLD participants had performances comparable to controls.

Overall, none of the DD children failed in all the oral language measures. However, some LD and a few NoLD dyslexic children had signs of actual oral language weakness with LD children, as a group, showing a greater weakness in tests of phonological working memory and in low-frequency word expressive vocabulary.

### Spelling assessment

Spelling abilities were tested through a standard *writing test* (Angelelli et al., 2008), composed of four sections (see Appendix):

Section A: *Regular words with complete one-sound-to-one-letter correspondence* (*N* = 70). Words were selected with different sources of phonetic-phonological complexity: (i) words made up of continuant sounds only (fricative, liquid or nasal consonants) vs. words also containing non-continuant (plosive) consonants; (ii) words made up only of consonant-vowel (CV) syllables vs. words also containing consonant clusters and doubled consonants; (iii) disyllabic vs. polysyllabic words. Different sources of phonetic-phonological complexity were used in order to determine variables influencing both segmentation and identification of phonemic string to be converted (for instance, continuant phones are, by nature, easiest to segment, and hence to identify, than non-continuant phones).
Section B: *Regular words requiring application of context-sensitive sound-to-spelling rules* (*N* = 10). In Italian, context-sensitive rules are required when spelling of a consonant depends on the following sound. For instance, the phoneme [k], is spelled C when followed by a consonant (e.g., CLIMA ([klima], climate) or by A, O, U (e.g., CASA [kaza], home), and CH when followed by E or I (e.g., BARCHE [barke], boats).
Section C: *Words with unpredictable transcriptions along phonological-to-orthographic conversion routine* (*N* = 55). This section includes: (i) words containing the phonemic group [kw], which in Italian may be transcribed by orthographic sequences QU, CU, or CQU; (ii) words containing syllables [tʃe], [ʃe], [dʒe], which may or may not require an I (e.g., [ʃentsa], science, is spelt SCIENZA and not ^*^SCENZA, while [ʃena], scene, is spelt SCENA and not ^*^SCIENA); (iii) words containing plosive phones followed by liquid consonants [r] which are homophones to their doubled pairs (e.g., FEBBRE, fever and not ^*^FEBRE, but LIBRO, book, and not ^*^LIBBRO); (iv) words containing segments [lj] —[ʎ] and [nj]—[ɲ], that are homophonous in most Italian variants to the extent that [biljardo/biʎardo], billiards, is spelt BILIARDO and not ^*^BIGLIARDO, while [folja/foʎa], leaf, is spelt FOGLIA and not ^*^FOLIA; similarly [opinjone/opiɲone], opinion, is spelt OPINIONE and not ^*^OPIGNONE, while [oɲuno/onjuno], everyone, is spelt OGNUNO and not ^*^ONIUNO.
Section D: *Non-words with one-sound-to-one-letter correspondence* (*N* = 25). Items were controlled for different sources of phonetic-phonological complexity, as were words in Section A: (i) continuance of sounds (non-words with continuant vs. non-continuant consonants); syllabic structure (non-words with consonant-vowel (CV) syllables vs. non-words also containing doubled consonants; and length (disyllabic vs. 3–4 syllable non-words). Similarly to Section A, phonetic/phonological variables are introduced in order to account for variables influencing acoustic-to-phonological analysis that is preliminary to an effective phonological-to-orthographic conversion procedure.

Words and non-words were given in separate sequences and in a single quasi-randomized order. The examiner read each item aloud in a neutral tone, i.e., without emphasizing presence of clusters, doubled consonants, or possible orthographic ambiguities. Children were asked to repeat each item before writing it down (so that the examiner could ensure that they had understood the item). When children failed to repeat or upon their request, the examiner read stimulus again. This occurred very seldom (about 1% of cases), and the second repetition was always sufficient to obtain a correct repetition of item. They were permitted to write in either capital or lower case letters. No feedback was provided on accuracy of written response. Final responses were counted, irrespective of correctness of first attempt. Children were tested individually.

The test has normative data for the 1st to the 8th grade (Angelelli et al., 2008).

### Data analysis

#### Spelling performance: quantitative analysis

Firstly, the number of correct spellings on each of the four sections of task was counted for every DD and control participant. We then computed the proportion of cases showing a clearly pathological performance on each of the four sections of task and on total. Following test norms, any performance below 1.5 *SD*s of test norms were considered pathological.

The number of correct spellings (expressed as a proportion of correct responses, i.e., percentage) were transformed by taking the arcsine of the square root of each data point in order to control for violations of ANOVA assumptions. However, for the sake of clarity, in the following paragraph we will refer to untransformed data, in particular to percentage of correct responses. Similarly, results and figures are based on percentage of correct responses.

The first ANOVA was performed on total spelling accuracy score, with group (LD, NoLD, and control participants) as between factor. A second ANOVA was performed on type of stimuli (regular words, context-sensitive words, unpredictable words, and non-words) as within factor and group (LD, NoLD, and control participants) as between factor.

Moreover, in order to specifically evaluate efficiency of phonetic-to-phonological analysis, we computed for each participant the percentage of correct responses in the various subsets of words (Section A, sub-sets 1–7) and non-words (section D, sub-sets 1–5). A MANOVA was performed to evaluate the influence of different sources of phonetic-to-phonological difficulties (see Appendix) on spelling accuracy among the three groups of children. In particular, in this analysis, group (LD, NoLD, and control participants) was entered as between factor, while lexicality (words, non-words) and presence of phonetic-to-phonological difficulties (present vs. absent) as within factors. Regarding phonetic-to-phonological difficulties, the MANOVA examined the effect of:

− Continuance of sounds (stimuli with continuant vs. non-continuant consonants). Operationally for words, we compared mean percentage of accuracy of subsets 1 + 3 + 4 vs. 5 + 6 + 7; for non-word subsets 1 + 3 vs. 4 + 5.
− Length (disyllabic vs. polysyllabic stimuli). Operationally for words, we compared mean percentage of accuracy of subset 1 vs. 2; for non-word subset 1 vs. 2.
− Presence of geminate consonants [stimuli made up by consonant-vowel (CV) syllables vs. stimuli containing doubled consonants]. Operationally for words, we compared mean percentage of accuracy of subset 1 vs. 4 and 5 vs. 7; for non-words we contrasted subset 1 vs. 3 and 4 vs. 5.

Interactions were explored through planned comparison. For significant effects and interactions we have reported effect sizes. In particular, we have reported partial eta squared in the case of ANOVAs and multivariate eta squared in the case of MANOVA. Note that for multivariate eta squared, reference values for small, medium, and large effects are considered to be 0.01, 0.06, and 0.13, respectively (Gall et al., 2011). For partial eta squared, reference points for a small, medium and large effect are 0.0099, 0.0588 and 0.1379 according to Cohen (1988).

#### Spelling performance: error analysis

An analysis was performed to identify nature of spelling errors, irrespective of section of test in which they emerged. Based on previous studies (Angelelli et al., 2004, 2008), errors were coded as:

*Phonologically plausible errors* (impaired spellings along lexical route): spelling errors that can be pronounced to sound like target words; these errors arise from over reliance on phoneme-to-grapheme conversion routine [e.g., ^*^CUOTA instead of QUOTA, (rate); ^*^FEBRE instead of FEBBRE, (fever), and remaining instances described in Section C of the spelling assessment];
*Phonologically non-plausible errors* (inaccurate spellings via sublexical routine): errors causing a change in phonemic makeup of a word reflecting difficulties in phonemic segmentation, phoneme-to-grapheme encoding, or a phonological/graphemic buffer disorder. This category included the following error subtypes:
*Errors based on minimal distance features:* substitutions of consonants or vowels with other consonants or vowels that differ in only one single distinctive feature [e.g., sonority, FINO (until) instead of VINO (wine); continuance, PESTA (crush) instead of FESTA (holiday)]. Doubling of a single consonant or dedoubling of a doubled consonant were also considered in this category;
*Other errors:* non-minimal-distance substitutions [e.g., ^*^BALO instead of BACO (worm)], omissions [e.g., ^*^VSONE instead of VISONE (mink)], insertions [e.g., ^*^MANRMO instead of MARMO (marble)] and letter transpositions [e.g., ^*^PATRO instead of PRATO (field)].
*Context-sensitive sound-to-spelling errors*: errors in application of context-sensitive sound-to-spelling rules [e.g., ^*^ADAGO instead of ADAGIO (slow) or SCEDA instead of SCHEDA (card)].

Spelling error profiles of each dyslexic participant (NoLD and LD) was computed and compared to reference data (Angelelli et al., 2008).

Moreover, an ANOVA was carried out with group (dyslexic NoLD, LD, and control participants) as between factor and error type (phonologically plausible, minimal distance, other errors, and context-sensitive sound-to-spelling errors) as within factor. Interactions were explored through planned comparison. As for quantitative data, analyses were performed on the arcsine of the square root of the proportion of errors (i.e., percentage/100); but, for the sake of presentation, figures, and means are based on untransformed data. For significant effects and interactions we have reported effect sizes (partial eta squared).

### Results: quantitative analysis

Individual spelling performances of both LD and NoLD dyslexic children are shown in Table 3. Seventy-nine percent of LD dyslexic children (11 out of 14) generally underperformed on total test score with respect to normative sample (Angelelli et al., 2008); while 93% (13 out 14) were poor in at least one subset of spelling task. Conversely, 8 out of 14 NoLD dyslexic children (57%) generally underperformed with respect to reference norms. Overall, 9 out of 14 children (64%) were poor in at least one subset of spelling task.

**Table 3:** **Individual spelling performances of dyslexic children with and without a history of language delay at the DDO spelling test (Angelelli et al., 2008)**.

	**Grade**	**Regular *N* = 70**	**Context-Sensitive *N* = 10**	**Unpredictable *N* = 55**	**Non-words *N* = 25**	**Total *N* = 160**
**NoLD DYSLEXIC CHILDREN**						
A.N.	3	49^*^	5^*^	25^*^	11^*^	90^*^
A.M.	3	48^*^	4^*^	24^*^	14^*^	90^*^
B.F.	3	52^*^	2^*^	23^*^	15^*^	92^*^
G.N.	3	69	10	42	22	143
C.Fi.	4	68	10	46	23	147
D.C.I.	4	69	9	45	25	148
L.V.	4	66^*^	8	36^*^	25	135^*^
M.A.	4	61^*^	5^*^	36^*^	17^*^	119^*^
N.A.	4	68	7^*^	36^*^	24	135^*^
S.C.	4	55^*^	7^*^	33^*^	14^*^	109^*^
S.N.	4	64^*^	6^*^	30^*^	20^*^	120^*^
B.S.	5	69	10	41	21^*^	141
C.Fr.	5	69	9	48	25	151
R.F.	5	70	9	47	24	150
*Mean*		62.64	7.21	36.57	20.00	126.43
*SD*		8.14	2.52	8.69	4.87	23.07
**LD DYSLEXIC CHILDREN**						
B.D.	3	48^*^	5^*^	19^*^	13^*^	85^*^
B.G.	3	64^*^	9	32^*^	22	127^*^
C.C.	3	43^*^	3^*^	19^*^	11^*^	76^*^
C.F.	3	58^*^	9	35	17^*^	119^*^
P.P.	3	49^*^	3^*^	24^*^	15^*^	91^*^
S.O.	3	53^*^	1^*^	21^*^	20	95^*^
B.F.	4	54^*^	4^*^	27^*^	16^*^	101^*^
C.G.	4	54^*^	6^*^	21^*^	15^*^	96^*^
C.V.	4	47^*^	5^*^	19^*^	12^*^	83^*^
L.C.	4	68	8	41	24	141
M.G.	4	49^*^	4^*^	27^*^	13^*^	93^*^
P.V.	4	57^*^	10	21^*^	12^*^	100^*^
Z.G.	4	66^*^	10	46	25	147
L.N.	5	69	6^*^	41	25	141
*Mean*		55.64	5.93	28.07	17.14	104.15
*SD*		8.36	2.87	9.34	5.10	22.39

*Regular: words with one-sound-to-one letter correspondence; Context-sensitive: regular words requiring the application of context-sensitive sound-to-spelling rules; Unpredictable: words with unpredictable transcription; non-words with one-sound-to-one letter correspondence. The asterisk marks participants whose performance was pathological with respect to standard reference data*.

**Figure 1:**
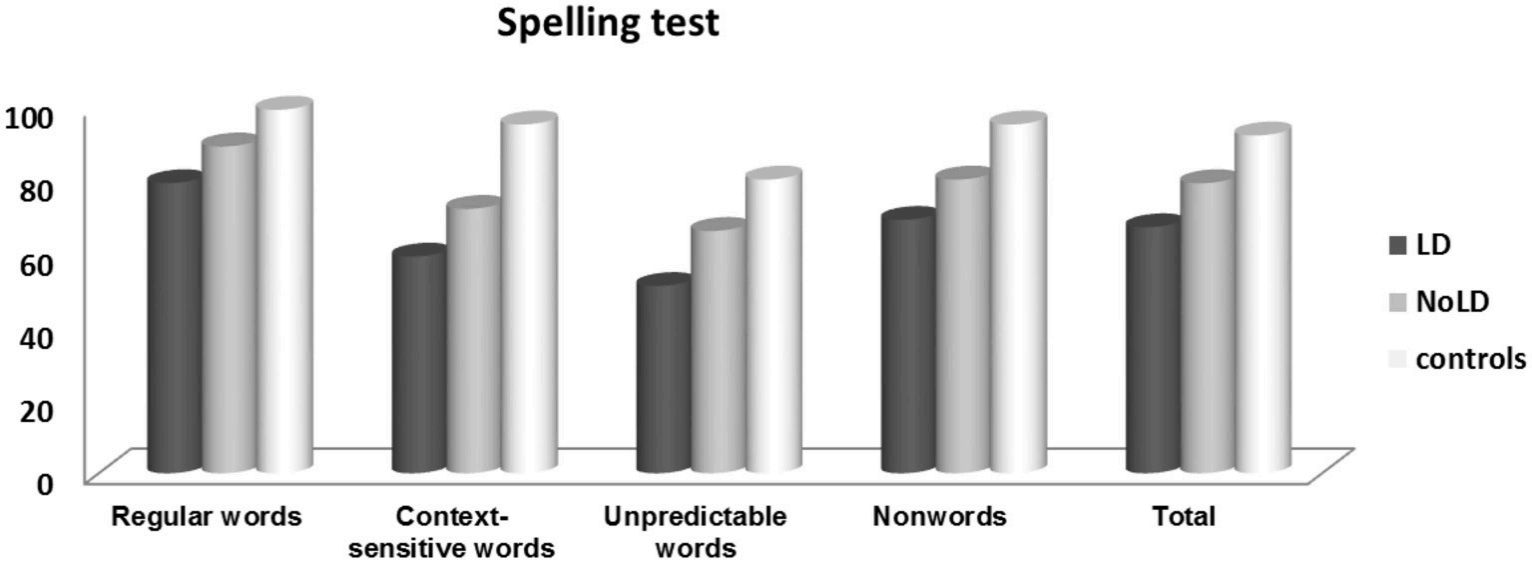
**Mean percentages of items correctly spelled by three groups of participants in total and in four sections of spelling test**.

Figure 1 reports mean percentages of items correctly spelled by the two groups of DD and control participants in the test as a whole and the four sections, respectively.

ANOVA on percentages of correct responses on whole test with *group* (LD, NoLD, and control participants) as unrepeated factor showed a significant effect of *group* [*F*_(2, 53)_ = 28.20; *p* < 0.0001; η^2^ = 0.52]: both groups of dyslexic children were poor spellers with respect to controls (at least *p* < 0.01), but children who suffered from a LD were significantly worse than NoLD ones (*p* < 0.01). A second ANOVA was performed on percentage of correct responses with *group* (LD, NoLD, and control participants) as between factor and *type of stimuli* (regular words, context-sensitive words, unpredictable words and non-words) as within factor. Main effects of *group* [*F*_(2, 53)_ = 28.39; *p* < 0.0001; ${}_{p}\eta^{2}=0.52$ and *type of stimuli* [*F*_(3, 159)_ = 47.50; *p* < 0.0001; ${}_{p}\eta^{2}=0.47$, and *group by type of stimuli* interaction [*F*_(6, 159)_ = 2.37; *p* < 0.05; ${}_{p}\eta^{2}=0.08$] were significant. Both groups of DD children (LD and NoLD) obtained lower performances in all categories of stimuli (at least *p* < 0.01) with respect to age-matched controls. However, LD children were poorer than NoLD in regular one-sound-to-one letter correspondence and unpredictable transcription words (at least *p* < 0.01), while no significant difference emerged for context-sensitive words and non-words.

The MANOVA exploring the influence of different sources of phonetic-to-phonological complexity in words and non-words transcription showed a main effect of *group* [λ = 0.49, *F*_(6, 102)_ = 7.40, *p* < 0.0001; multivariate η^2^ = 0.89], *lexicality* [λ = 0.69, *F*_(3, 51)_ = 7.75, *p* < 0.001; multivariate η^2^ = 0.53], and *phonetic-to-phonological difficulties* [λ = 0.67, *F*_(3, 51)_ = 8.55, *p* < 0.0001; multivariate η^2^ = 0.56]. LD and NoLD dyslexic children were less accurate than age-matched controls (97.2%; at least *p* < 0.001), and LD children (73.8%) did poorer than NoLD ones (84.4%; *p* < 0.05). All children had lower performance with non-words respect to words (80.9 vs. 89.4% of accuracy respectively) and with items containing phonetic-to-phonological difficulties respect to the others (84.7 vs. 85.6%, respectively). In addition, the *group* by *phonetic-to-phonological difficulties* interaction was significant [λ = 0.72, *F*_(6, 102)_ = 3.04, *p* < 0.01; multivariate η^2^ = 0.63]. Exploration of the interaction showed that NoLD dyslexic children were affected by the presence of phonetic-to-phonological difficulties in a similar measure as age-matched controls; this result was confirmed also by univariate analysis for each dependent variables. Conversely, LD dyslexic children performance was modulated to a larger extent by phonetic-to-phonological difficulties than controls (*p* < 0.0001), for each dependent variables examined (at least *p* < 0.05). Moreover, LD dyslexic children were more affected by the presence of phonetic-to-phonological difficulties than NoLD dyslexic children (*p* < 0.001): the presence of doubled consonants produced a greater reduction in accuracy among LD with respect to NoLD children. In fact, the presence of doubled consonants produced a reduction of spelling accuracy (respect to the condition without doubled consonants) by 36.3% among LD children, 9.5% among NoLD children, while the effect of doubled letters was negligible in controls. Note that the accuracy of LD children in spelling stimuli with doubled consonants was very low (51.6%) Presence of non-continuant sounds produced a reduction of accuracy by 4.1% among LD children, while the same effect was negligible between both NoLD and control children. The difference in accuracy between long and short stimuli was 10.7% among LD children, while in NoLD children and controls was 3.6 and 2.9%, respectively.

### Error analysis

In order to clarify better the nature of spelling deficit in the two DD groups, analysis of error type produced was performed. Table 4 shows error rates of each LD and NoLD dyslexic participant for critical categories. Following test norms, a rate of error exceeding by 1.5 standard deviations the normative sample was considered pathological. Analysis of error profiles indicates that LD dyslexic children, with respect to NoLD children, often had higher pathological error rates. LD dyslexic children were mainly pathological for simple phoneme-to-grapheme conversion errors (other errors; 93%) and minimal distance errors (71%), followed by phonologically plausible errors and context-sensitive errors (about 64% in both cases). Conversely, NoLD dyslexic children produced a higher rate of simple phoneme-to-grapheme conversion errors (71%) and phonologically plausible errors (57%), whereas only a minority of children also produced high rates of minimal distance errors (36%).

**Table 4:** **Error rates of NoLD and LD dyslexic participants**.

	**ERRORS**			
	**Phonologically plausible**	**Minimal distance**	**Other**	**Context-sensitive**
**NoLD DYSLEXIC CHILDREN**				
A.N.	18^*^	36^*^	17^*^	5^*^
A.M.	26^*^	30^*^	8^*^	9^*^
B.F.	18^*^	32^*^	13^*^	18^*^
G.N.	8	3	7^*^	0
C.Fi.	9	3	1	0
D.C.I.	10	0	1	1
L.V.	12	5	9^*^	2
M.A.	16^*^	11^*^	8^*^	6^*^
N.A.	15^*^	0	7^*^	3^*^
S.C.	17^*^	28^*^	15^*^	1
S.N.	15^*^	6	20^*^	7^*^
B.S.	14^*^	2	4^*^	0
C.Fr.	8	0	0	1
R.F.	8	1	0	1
*Mean*	13.86	11.21	7.86	3.86
*SD*	5.14	13.72	6.47	4.99
**LD DYSLEXIC CHILDREN**				
B.D.	20^*^	36^*^	20^*^	11^*^
B.G.	15	6	14^*^	3
C.C.	23^*^	38^*^	17^*^	19^*^
C.F.	10	21^*^	15^*^	1
P.P.	13	29^*^	24^*^	13^*^
S.O.	19^*^	13^*^	34^*^	13^*^
B.F.	23^*^	26^*^	10^*^	6^*^
C.G.	27^*^	26^*^	11^*^	4^*^
C.V.	24^*^	50^*^	8^*^	1^*^
L.C.	9	5	5^*^	1
M.G.	23^*^	32^*^	7^*^	7^*^
P.V.	24^*^	21^*^	29^*^	1
Z.G.	9	3	3^*^	0
L.N.	13^*^	0	2	4^*^
*Mean*	18.00	21.86	14.21	6.00
*SD*	6.31	14.91	9.71	5.86

*Asterisk marks participants whose error rate is more than controls standard reference data*.

**Figure 2:**
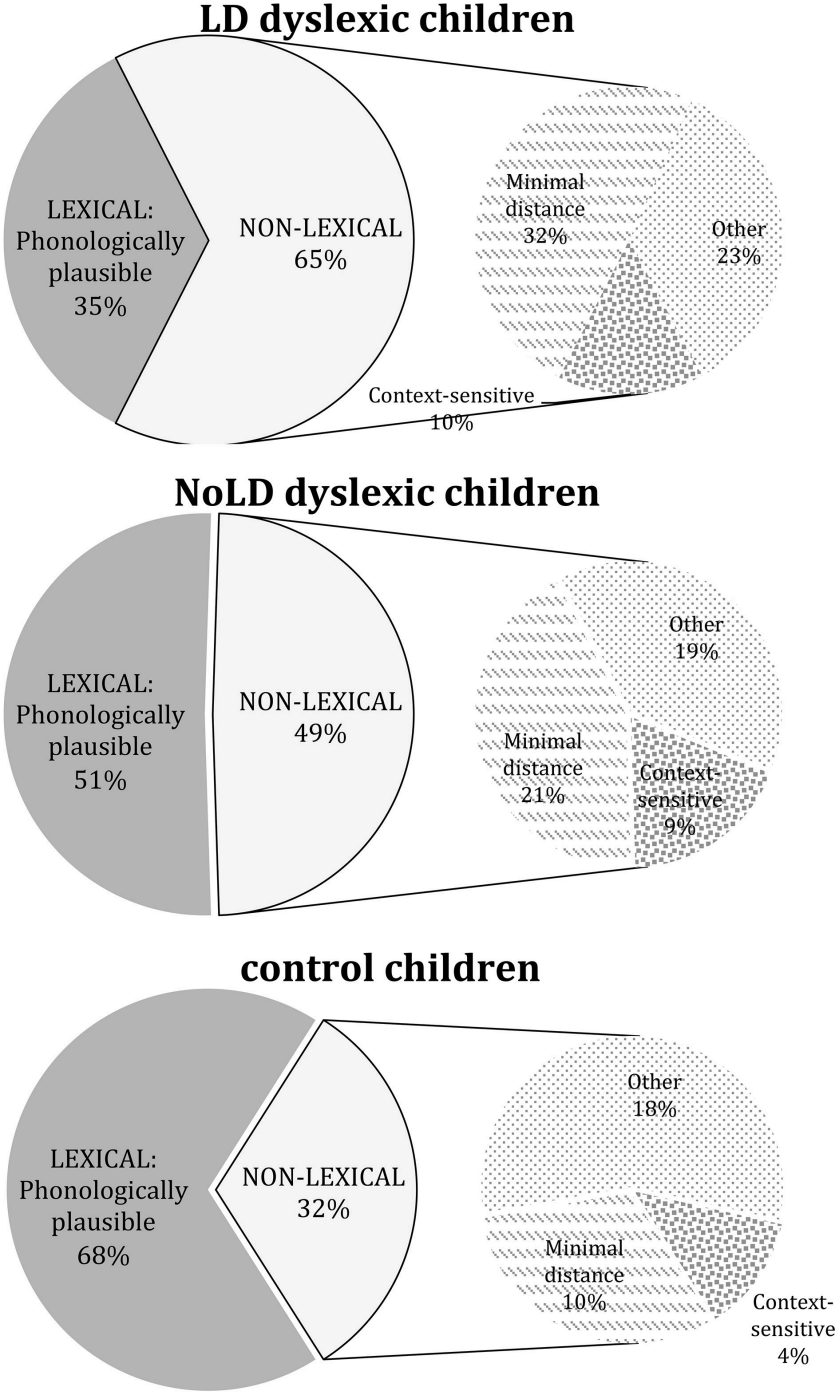
**Left side of figures reports the percentage of lexical and non-lexical errors, respectively, in LD, NoLD dyslexic children, and control participants**. Right side of figures represents the proportion of minimal distance, other and context sensitive errors among non-lexical errors in the three groups of participants.

Figure 2 (left side) reports percentage of errors reduced into two main categories (coherent with Notarnicola et al., 2012): lexical errors (phonologically plausible errors) vs. all non-lexical ones (context-sensitive, minimal distance and other errors), whose relative percentages are indicated on the right side of figure.

Two-way ANOVA with *group* (LD, NoLD, and control participants) as unrepeated factor and *error category* (phonologically plausible, context-sensitive, minimal distance and other errors) as repeated factor showed a significant effect of *group* [*F*_(2, 53)_ = 13.03; *p* < 0.0001; ${}_{p}\eta^{2}=0.33$], *error type* [*F*_(3, 159)_ = 62.62; *p* < 0.0001; ${}_{p}\eta^{2}=0.54$], and interaction of *group x error type* [*F*_(6, 159)_ = 8.68; *p* < 0.0001; ${}_{p}\eta^{2}=0.25$]. LD dyslexic children had a higher rate of all types of errors (except for other errors) with respect to control children (at least *p* < 0.01 in all comparisons), and a smaller rate of phonologically plausible errors associated with a higher rate of minimal distance misspellings with respect to NoLD children (at least *p* < 0.05).

A second ANOVA was performed in order to compare error profile of three groups (LD, NoLD, and control participants) with errors reduced into lexical vs. non-lexical errors. Only interaction *group x error type* was significant [*F*_(2, 53)_ = 19.75; *p* < 0.0001; ${}_{p}\eta^{2}=0.43$]. Exploration of interaction showed that, while in NoLD group there was a comparable percentage of lexical and non-lexical errors (51 vs. 49%, respectively), in LD dyslexic children non-lexical errors were significantly more prevalent than lexical ones (65 vs. 34%, *p* < 0.01). Note that in control children, the pattern was the opposite: a larger percentage of lexical errors with respect to non-lexical one (68 vs. 32%; *p* < 0.0001).

## Discussion

The aim of the study was to determine whether DD children with a history of language delay presented a specific pattern of spelling impairment, compatible with a defective orthographic lexical acquisition, already documented in Italian dyslexic-dysgraphic children, associated with persistent phonological spelling difficulties.

Analysis of oral language abilities showed that LD dyslexic children presented, as a marker of previous language delay, a concurrent weakness in phonological processing, revealed by an impaired performance in working memory (especially for phonologically similar words), and a deficit in expressive vocabulary (especially for low-frequency words). These results confirm other data from previous studies of our group, showing that subtle oral language deficits may persist in children with previous LD, though no longer clinically apparent at school age (Chilosi et al., 2003, 2009; Brizzolara et al., 2006).

Regarding written language deficits, while no differences in severity and characteristics of reading deficit emerged between DD children with LD and NoLD, confirming previous findings (Chilosi et al., 2003, 2009; Brizzolara et al., 2006), analysis of spelling impairment disclosed quantitative and qualitative differences between groups.

In general, both groups of DD children were poor spellers with respect to controls. This result was expected based on previous research (Chilosi et al., 2003; Angelelli et al., 2004, 2010a; Chilosi et al., 2009). However, LD dyslexic children presented a differential pattern of impairment: in addition to more severe spelling difficulties on words with unpredictable transcription, they were also poorer on regular stimuli presenting specific sources of phonetic-to-phonological complexity. Poor performance on items requiring lexical knowledge is in line with other studies on Italian dyslexic-dysgraphic children (Angelelli et al., 2004, 2010a) showing a defective development of orthographic lexicon, that, in the case of LD children, may seem more severe. Poor lexical spelling was found to characterize long-term spelling outcomes in Italian adolescents with a history of specific language impairment (Brizzolara et al., 2011). Regarding performance on regular stimuli with one-sound-to-one-letter correspondence, LD children were significantly more affected than NoLD ones by the phonological complexity of stimuli (especially presence of doubled consonants but also non-continuant sounds and length). Greater spelling difficulties with stimuli containing phonetic-to-phonological difficulties are coherent with fragility of acoustic-to-phonological conversion. Non-continuant phones are more difficult to segment and identify than continuant ones. Predominant misspellings consisting of devoicing of voiced consonants and doubling of single (or dedoubling of doubled consonants) have been described in Italian brain-damaged patients with acquired dysgraphia and ascribed to acoustic-to-phonological deficits (Luzzatti et al., 2000). Also length is a variable of complexity along sublexical procedure (Angelelli et al., 2014): The longer the phonological string (to be transcribed), the higher the number of possible error loci in the two conversion processes (i.e., acoustic-to-phonological and phonological-to-orthographic) that select phonological and graphemic elements, and the heavier the demand on two memory buffers (phonological and graphemic buffers). Coherent with length spelling effect, difficulties in managing strings of increasing longer phonologically similar words emerged in working memory evaluation of LD children.

Overall, quantitative analysis showed that LD dyslexic children suffered from a defective use/acquisition of lexical spelling procedure together with a concomitant sublexical deficit, highlighted by greater difficulties with respect to NoLD children in spelling stimuli conveying phonological difficulties.

Qualitative error analysis confirmed this pattern of results. LD dyslexic children shared with NoLD children a high rate of phonologically plausible errors, indicative of a prevalent reliance on sublexical processing. In fact, reliance on phonology may produce errors when a lexical representation is necessary to solve spelling ambiguities. However, the sublexical spelling procedure may not be efficient. When simple conversion rules were applied, LD dyslexic children committed a high number of substitutions of consonants or vowels with other consonants or vowels that differs only in one single acoustic-to-phonological distinctive feature. This pattern of errors was more frequent in LD than in NoLD dyslexic children. Presence of minimal distance misspelling errors indicates a specific fragility of minimal distinctive features when processed along sublexical spelling procedure. Such a weakness, in turn, may be ascribed to difficulties along phonetic/phonological analysis. Given this evidence, it seems that analysis of spelling performance in our study may have captured fragility in processing subtle phonetic-to-phonological differences in children with dyslexia and previous LD.

Since we did not directly test auditory analysis, present data do not permit us to discriminate between these alternatives and further research is required to address this issue. However, a recent study by Ziegler et al. (2015) found that children with SLI had poorer than normal consonant identification when measured in ecologically valid conditions (speech perception–in-noise-measure). In particular, identification of all phonetic features was impaired, although the deficit was stronger for voicing perception. SLI children experienced normal “release from masking” (better identification in fluctuating than in stationary noise), interpreted by the authors as a central deficit in feature extraction rather than a deficit of low-level capacities, due to an inefficient mapping of acoustic into phonetic features. Moreover, speech intelligibility deficits predicted phonological deficit in non-word reading.

Finally, the generally worse performance of LD dyslexic children is coherent with other previous finding, although comparisons with other studies are difficult due to methodological differences (McCarthy et al., 2012). Chilosi et al. (2003) studied Italian DD children with and without language delay and found LD children less accurate than NoLD children in transcription of a meaningful text and isolated word and non-word items. McCarthy et al. (2012) examined spelling performance in SLI children, children with DD and those with both SLI and dyslexia. Results showed that children with SLI performed similarly to their typically developing peers, whereas children with concurrent SLI and DD showed clear spelling deficits, although no difference was detected between the two groups of DD children. Finally, Bishop and Clarkson (2003) studied writing performance of children with SLI, unaffected co-twin, and co-twin with a history of SLI (but no longer evident deficits) with two writing tasks (a word spelling-to-dictation task and a written-narrative task). The authors found that not only SLI children underperformed on spelling tasks but also the resolved group performed poorly. Coherent with this study, error analysis showed that phonological errors were high in both SLI children and those with a history of SLI, with respect to control children.

In conclusion, results showed that both groups of DD children suffered from developmental dysgraphia, confirming a high incidence of spelling deficits in children with DD, also in languages with regular sound-to-spelling mapping (Wimmer and Mayringer, 2002; Angelelli et al., 2004, 2010a,b; Marinelli et al., 2009). Previous data on Italian dyslexic-dysgraphic children (e.g., Angelelli et al., 2004, 2010a), found a prevalence of surface dysgraphia, with impaired spelling along lexical orthographic procedure. However, children with a previous language delay exhibited a concomitant sublexical spelling deficit, with a specific fragility on minimal distinctive traits, suggesting an impaired auditory analysis and/or phonological encoding defect.

## Ethics statement

The study was conducted according to the principles of the Helsinki Declaration and was approved by the local committee of the Department, by school authorities and by IRCCS STELLA MARIS committee.

## Author contributions

All authors listed, have made substantial, direct and intellectual contribution to the work, and approved it for publication.

### Conflict of interest statement

The authors declare that the research was conducted in the absence of any commercial or financial relationships that could be construed as a potential conflict of interest.

## Appendix

Subtests of the spelling task (Angelelli et al., 2008).

**Table:** **(A) Regular words with one-sound-to-one-letter correspondence (*n* = 70)**.

	**Examples (translation)**	**Continuance**	**Cluster**	**Doubled consonants**	**Syllables**	***N***
1	Sole (sun)	Yes	No	No	2	10
2	Lavoro (work)	Yes	No	No	3/4	10
3	Senso (sense)	Yes	Yes	No	2	10
4	Valle (valley)	Yes	No	Yes	2	10
5	Dito (finger)	No	No	No	2	10
6	Prato (field)	No	Yes	No	2	10
7	Tappo (cork)	No	No	Yes	2	10

**Table:** **(B) Regular words (syllabic conversion rules) (*n* = 10)**.

	**Examples**	**Rule**	***N***
8	gola/ghiro/valigia (throat/dormouse/suitcase)	k, g, tS, dJ	10

**Table:** **(C) Words with unpredictable transcription (*n* = 55)**.

	**Examples**	**(translation)**	**Ambiguity**	***N***
9	scena/scienza	(scene/science)	[tʃe],[ʃe], [dʒe] ± i	10
10	paglia/balia	(straw/nurse)	[ʎ]: GL/LI	10
11	segno/genio	(sign/genius)	[ɲ]: GN/NI	10
12	libro/febbre	(book/fever)	BR/BBR	10
13	cuore/quota/aquila	(heart/share/eagle)	[kw]: CU/QU	15

**Table:** **(D) Non-words with one-sound-to-one-letter correspondence (*n* = 25)**.

	**Examples**	**Continuance**	**Cluster**	**Doubled consonant**	**Syllables**	***N***
1	nise	Yes	No	No	2	5
2	vimàne/ramàsola	Yes	No	No	3/4	5
3	seffa	Yes	No	No	2	5
4	tido	No	No	Yes	2	5
5	nitta	No	No	No	2	5
